# Extracting Gene Networks for Low-Dose Radiation Using Graph Theoretical Algorithms

**DOI:** 10.1371/journal.pcbi.0020089

**Published:** 2006-07-21

**Authors:** Brynn H Voy, Jon A Scharff, Andy D Perkins, Arnold M Saxton, Bhavesh Borate, Elissa J Chesler, Lisa K Branstetter, Michael A Langston

**Affiliations:** 1 Life Sciences Division, Oak Ridge National Laboratory, Oak Ridge, Tennessee, United States of America; 2 Department of Computer Science, University of Tennessee, Knoxville, Tennessee, United States of America; 3 Department of Animal Science, University of Tennessee, Knoxville, Tennessee, United States of America; The University of Texas Southwestern, United States of America

## Abstract

Genes with common functions often exhibit correlated expression levels, which can be used to identify sets of interacting genes from microarray data. Microarrays typically measure expression across genomic space, creating a massive matrix of co-expression that must be mined to extract only the most relevant gene interactions. We describe a graph theoretical approach to extracting co-expressed sets of genes, based on the computation of cliques. Unlike the results of traditional clustering algorithms, cliques are not disjoint and allow genes to be assigned to multiple sets of interacting partners, consistent with biological reality. A graph is created by thresholding the correlation matrix to include only the correlations most likely to signify functional relationships. Cliques computed from the graph correspond to sets of genes for which significant edges are present between all members of the set, representing potential members of common or interacting pathways. Clique membership can be used to infer function about poorly annotated genes, based on the known functions of better-annotated genes with which they share clique membership (i.e., “guilt-by-association”). We illustrate our method by applying it to microarray data collected from the spleens of mice exposed to low-dose ionizing radiation. Differential analysis is used to identify sets of genes whose interactions are impacted by radiation exposure. The correlation graph is also queried independently of clique to extract edges that are impacted by radiation. We present several examples of multiple gene interactions that are altered by radiation exposure and thus represent potential molecular pathways that mediate the radiation response.

## Synopsis

Microarrays take snapshots of gene expression across the genome. Many versions of clustering techniques have been developed in an effort to classify large scale microarray datasets into smaller sets of genes with shared expression patterns. These efforts have been motivated in part by the concept that genes with shared expression patterns are more likely to exhibit correlated expression levels than do genes with unrelated functions, and thus clusters of co-regulated genes hold insight into identifying genes that function in common cellular pathways. Voy and colleagues have developed a novel means of clustering built upon graph algorithms that extracts sets of perfectly interconnected genes—cliques—from graphs built from gene expression data. Cliques and other tools are used to identify relationships in expression between multiple genes. They illustrate this method by applying it to the study of low-dose radiation exposure in mice and in the process identify a variety of relationships that are activated in spleen by low levels of radiation exposure.

## Introduction

“Guilt-by-association,” the assumption that genes with similar expression patterns participate in common cellular functions, drives a growing body of effort to extract cellular pathways from microarray data [1–4]. The general tenet is that genes encoding proteins participating in a common pathway will display correlated expression levels when analyzed at sufficient scale, and that the identities and known functions of these genes can be used to highlight existing and assimilate new functional pathways. A number of recent studies validate the concept of guilt-by-association, demonstrating that genes co-expressed across multiple conditions are more likely to represent common functions than would be expected by chance alone [5,6]. To date the computational methods to extract such patterns lag far behind the general agreement about their utility.

The majority of methods to extract pathways of co-regulation from microarray data begin with a measure of similarity—e.g., Euclidean distance, Pearson's correlation coefficient—that describes the degree to which expression levels between pairs of genes are correlated across multiple conditions [7]. The matrix of correlations across the microarray, typically representing the pairwise similarity of the expression patterns of thousands of genes, is the starting point from which to organize genes into clusters. Clustering includes a wide variety of algorithms for organizing multivariate data into groups with approximately similar expression patterns, and a wealth of clustering approaches has been proposed [8]. However, there are several important limitations to the vast majority of clustering algorithms that are in contrast to the reality of biology. The first is that they are disjoint, requiring that a gene be assigned to only one cluster. While this simplifies the amount of data to be evaluated, it places an artificial limitation on the biology under study in that many genes play important roles in multiple but distinct pathways. The other main problem is that most measures of similarity used in clustering algorithms do not permit the recognition of negative correlations, which are also common and equally meaningful.

As an alternative to assigning genes to clusters, the correlation matrix can be thresholded to create a graph comprised only of edges (gene-gene correlation values) whose weights exceed a predefined value. Allocco and colleagues originally described such graphs as relevance networks [9]. In a relevance network, both positive and negative correlations exceeding a specified threshold are retained and displayed graphically, allowing visual recognition of highly connected subsets of genes. Recent studies have mined relevance networks to extract co-expressed genes in cancer cells [10,11] and myopathic muscle biopsies [12]. While those efforts provided gene subsets of biological relevance to the respective conditions, they were limited to pairwise relationships that could be extracted manually from the graphs. Relevance networks contain many dense sub-graphs of tightly interconnected gene sets that intuitively represent the greatest potential for identifying members of common pathways. Without a systematic means to extract the aggregate relationships between multiple genes, however, many of the most interesting relationships remain embedded within the web of correlations.

We have developed a computational approach that exploits graph theoretical algorithms to identify comprehensively the tightly connected subsets of genes present in relevance networks. In the most extreme case, in which a sub-graph contains all possible edges between vertices in the sub-graph, this structure is called a clique. In terms of gene expression, clique represents the most trusted potential for identifying a set of interacting genes. Solving clique, however, is a nondeterministic polynomial-complete problem, and a classic graph-theoretic problem in its own right [13]. We have previously developed novel graph algorithms that employ vertex cover and allow clique to be solved in polynomial time [14–17]. Recently we applied these algorithms to identify cliques of co-expressed genes as part of an effort to annotate quantitative trait loci associated with neural function [18]. Here we extend these algorithms to identify differential gene relationships, i.e., gene-gene interactions that are induced or repressed by a specific treatment. We illustrate our approach using a set of microarray data that was generated from spleen of mice exposed in vivo to low-dose ionizing radiation (IR). Radiation is a well known agent of DNA damage at relatively high but sub-lethal doses[19]. The response to lower doses, however, such as those received from medical imaging, radiotherapy and occupational exposures, is poorly defined and largely dependent upon genetic background [20]. The data used herein were derived from a study that explored the role of genetic susceptibility in the response to IR. Six strains of inbred laboratory mice were exposed to 10 cGy X-rays in vivo, after which gene expression changes in spleen were profiled using microarrays. We describe our graph theoretical-based toolchain for identifying overlapping subsets of genes with tightly correlated expression levels and demonstrate the biological insight that this method provides.

## Results

### Calculation of the Correlation Matrix

Microarray data used in this study were collected as part of an effort to examine the effects of genetic background on the response to ionizing radiation in multiple mouse tissues. A panel of six common inbred strains of laboratory mice were exposed to a single acute dose of 10 cGy X-ray, and tissues were harvested 3.5 hr after irradiation for microarray gene expression profiling. Data analyzed in this effort were restricted to spleen, which is a target of the immunological effects of radiation exposure. RNA samples from sham-irradiated (controls) and irradiated mice were randomly paired to form biological replicates (a minimum of three per mouse strain). Each microarray hybridization consisted of one biological replicate, in which the control and irradiated samples were labeled with either Cy3 or Cy5 fluorescent dyes and hybridized to a single array. Each biological replicate was hybridized in duplicate, swapping the dyes to control for potential dye-specific effects. Both genetic variation between the six inbred strains and inter-individual variation within strains drive non-zero correlations in the graphs described below. An overview of the experimental design and microarray hybridizations is depicted in Figure S1. Complete results of the biological response and differential expression results will be reported elsewhere (B. Voy, unpublished data), including complete access to the primary microarray data through the Gene Expression Omnibus (ncbi.nlm.nih.gov/geo/).

**Figure 1:**
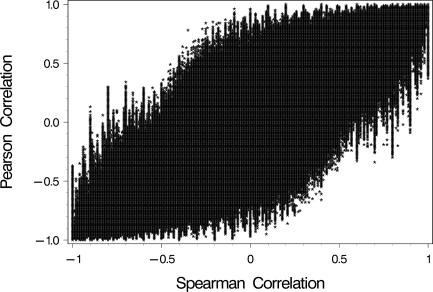
Comparison of Pearson's and Spearman's Correlation Coefficients

**Figure 2:**
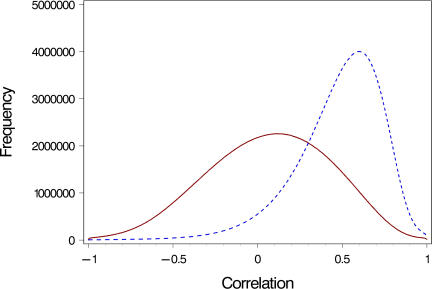
Impact of Normalization on the Correlation Distributions Normalization results in a distribution approximately centered around zero.

**Figure 3:**
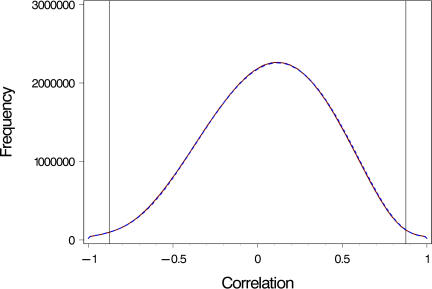
The Distributions of Correlations in Control and IR Are Highly Similar Control (blue dashed) and IR (red solid) lines overlay each other across the entire distribution. The vertical lines in each tail of the distribution delineate the edges that were included in the graph after applying the threshold of |0.875| to the correlation matrix.

Our first step in using graph theory to extract gene networks was to create a correlation matrix across the entire set of microarray data. A total of 21,547 mRNAs were represented on the microarray platform used (Compugen Mouse OligoLibrary, 2.0; http://www.labonweb.com), resulting in a correlation matrix of ~200,000,000 values. Although we describe our approach as applied to data from long oligonucleotide arrays, our method is equally relevant for data from Affymetrix or other array platforms, or for other types of high throughput, quantitative data. To explore the relative utility of Spearman's rank versus Pearson's correlation coefficients for our purposes, we calculated the correlation matrix using each metric and plotted the distributions. Pearson's is preferred as it utilizes information in the data more fully, but Spearman's would be less sensitive to the noise typically seen in array data [21]. As shown in Figure 1, the relationship between the two coefficients was weak, and for a given Spearman correlation, Pearson coefficients had a wider range of values, producing vertical spikes in Figure 1. These reflect the greater information in Pearson's correlations, and since no strong evidence of deviations from normality were found, Pearson's correlations were used for all further analyses. To confirm the efficacy of between slide normalization procedures we plotted the distribution of Pearson's coefficients for the control data before and after normalization, as shown in Figure 2. It is expected that the majority of genes are not correlated at all, and that the center of the distribution should approximate zero. Normalization effectively shifted the distribution to one centered very close to zero with a slight positive shift, which we predict reflects a slight degree of positive correlation due to technical measures. We then plotted the distribution of correlations for control and IR separately, to determine if they exhibited the same characteristics. As shown in Figure 3, the two distributions were highly similar, as evidenced by the near overlap of the distribution plots.

### Distribution of the Matrix and Threshold Selection

From the resultant matrix we wanted to create a graph representing the strongest—and most likely to reflect biological meaning—relationships between genes. Weighted graphs produced from this type of data consist of vertices representing genes and edges whose weights are indicative of the correlation between each pair of vertices (genes). Given a suitable threshold, *t*, edges with weights less than *t* are discarded; edges with weights at least *t* are retained. This produces an unweighted graph, *G*, whose structural properties are of interest. Creating the graph requires selecting a threshold correlation value, above which all edges will be included in the graph. Our main criterion was to select a value that would largely represent true expression-based correlations and would not be influenced by non-specific signal from the arrays. To assess this, we recreated the correlation matrix, including the background-subtracted signal values for a series of nonspecific buffer spots distributed across the arrays. These spots tend to give a signal value that is approximately 10% above background, and they are not included in analysis of differential expression. We determined the numbers of edges connecting these spots as vertices across a range of threshold correlation values. Correlation values with salt spots dropped markedly as the threshold value increased, and very few correlations exceeded 0.875 (0.38%, a total of 162 out of 42,194 correlations), indicating that using this as a threshold for creating a graph would largely exclude any nonspecific correlations. From a statistical significance viewpoint, if we choose *p* = 0.01 to indicate biologically real correlations, and correct this for multiple testing by dividing by 21,754 genes on the arrays, a standard normal critical value of 5.042 results. Applying Fisher's z-transformation in reverse, this corresponds to a correlation of 0.85 (*n* = 19 minimum). Therefore our threshold of 0.875 will give edges in the resultant graphs representing only statistically significant correlations (*p* < 0.01).

Table 1 displays the statistics of edges included in the graph using the 0.875 threshold value. For control, 0.061% of all possible correlations were included in the graph, with a slightly higher percentage (0.068%) included in the graph for IR. Degree refers to the number of other vertices with which a gene is connected. The average degree for a vertex in controls was 18 other genes, which increased to 20 in IR. The mode for degree equaled 1 in both control and IR, indicating that most genes in the graph had very low connectivity. The vast majority of edges in each graph were positive (91% in control and 91.5% in IR), indicating that expression levels for most pairs of genes changed in parallel rather than inversely. Most genes that did display inverse correlations with other genes had only one or two such negative edges, reflected by a median degree equal to 1 for negative edges in controls and 2 in IR.

**Table 1:**
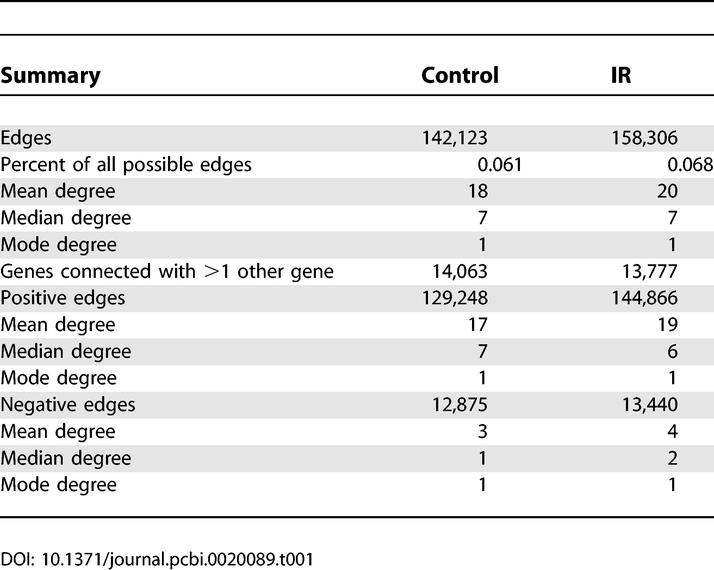
Summary of Edges Included in the Graphs

### Computation of Clique

**Figure 4:**
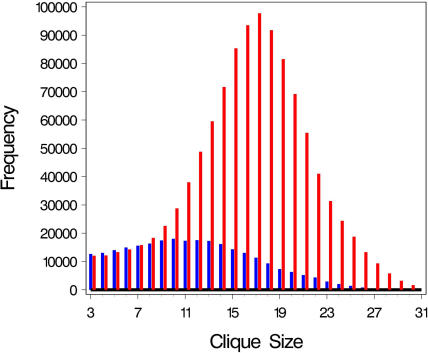
Distribution of Clique Sizes in Control and IR Maximum clique and average clique sizes were larger in IR (red bars) than control (blue bars).

**Figure 5:**
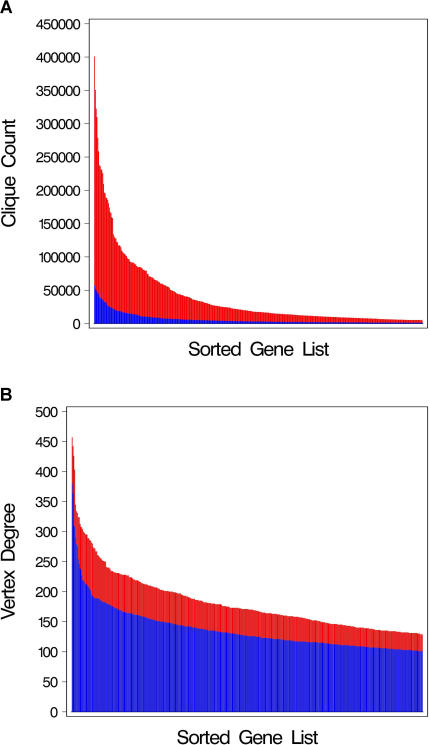
Scale Free Properties of Gene Connectivity Gene lists were sorted in order of abundance for each condition, and the 400 genes most abundant in control (blue bars) and IR (red bars) were plotted against clique membership (A) and vertex degree (B). Although average vertex degree and clique membership were not markedly different between control and IR, the genes most abundant in IR cliques were more highly connected and present in more cliques than in control.

The output of thresholding the correlation matrix was an edge-weighted graph comprised of the tightest relationships between genes. Many types of *k*-dense sub-graphs existed within this graph, but we sought to extract cliques as the most intuitively interesting structure in terms of biological relationships. Clique in an undirected graph *G*, is a set of vertices V such that for every two vertices in V, there exists an edge connecting the two. In particular, we solved the maximum clique problem, the goal of which is to find the largest *k* for which *G* contains a clique of size *k*, that is, a sub-graph isomorphic to *K_k_*, the complete graph on *k* vertices [13]. The importance of *K_k_* lies in the fact that each and every pair of its vertices is joined by an edge in *G*. Figure 4 shows the distributions of cliques by size for both control and for IR. As is apparent from this graph, clique size (number of genes in a clique) tended to be larger in IR than in control (control = 12, IR = 16; mean size). The number of cliques was considerably greater in IR (*n* = 1,079,156) than in control (*n* = 268,611), as detailed in Table 2. We predict that this is due to the fact that variance in expression was significantly greater in data from the IR group compared to controls (unpublished data), which served to drive more non-zero correlations in IR. Approximately half of all genes on the arrays were involved in cliques in both control and in IR (11,233, control; 10,846, IR). Like the worldwide web and many other networks, biological networks are predicted to be scale-free, with most vertices connected to few or no others, and with a smaller subset of vertices displaying high connectivity [22–25]. This feature is illustrated in Figure 5, depicting the numbers of cliques and the degree according to gene. As shown in Table 2, only about 1.7 % of all genes on the arrays were involved in more than 0.5% of cliques for both control and for IR.

**Table 2:**
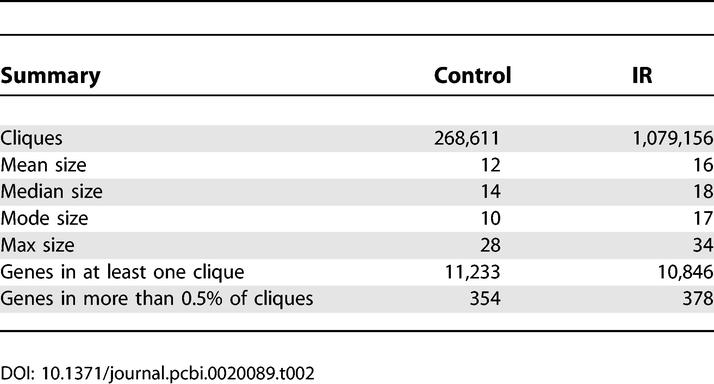
Clique Summary Statistics

Our overall goal is to develop a method that uses microarray data to identify genes involved in shared cellular pathways as a means to gain additional understanding about a biology of interest. Therefore we next queried both the graphs and the cliques, asking a series of questions about the relationships between genes that were altered by radiation exposure. We illustrate below several ways in which the graph can be queried to identify relationships between genes that respond to a specific condition, in this case exposure to IR. We refer to this overall approach as differential clique analysis.

### Differentially Expressed Genes

Mixed model analysis of the spleen IR dataset revealed that many differentially expressed genes were involved in the immune response and inflammation, consistent with biological effects of radiation [26,27], while others had little or no functional annotation. We applied guilt-by-association by selecting genes from the latter group and then filtering the clique lists to identify those with a high degree of connectivity in radiation but not controls. As an example, latent transforming growth factor beta binding protein 2 *(Ltbp2)* expression was significantly down-regulated by IR. Ltbp2 is a member of a family of proteins so named because of their ability to bind and regulate the availability of transforming growth factor beta (TGF-β), a hormone that orchestrates the cellular response to DNA damage after IR [28]. Unlike other Ltbp family members, Ltbp2 may not bind TGF-β but rather may play a structural role by integrating with elastin containing microfibrils in the cortex of the spleen [29,30]. Virtually nothing is known about its role in the radiation response. *Ltbp2* was represented in 573 times as many cliques in IR than in control. It shared edges with 13 genes in control but with 119 in dose (seven of which were in common between the two conditions), indicating that IR activated connections between *Ltbp2* and many other genes. We then used Gene Ontology (GO) analysis to determine if the set of 112 genes connected to *Ltbp2* only in IR were enriched in any specific biological functions, which would provide additional insight into its role in the IR response. Of the 112 genes, 54 were annotated with a GO term(s) in the category of molecular function and thus amenable to GO analysis. This subset of 54 genes was significantly enriched for the GO categories GTPase activator activity (three genes; *p* = 0.030) and marginally for the category of structural molecule activity (six genes; *p* = 0.055), consistent with a predicted role for Ltbp2 in maintaining the structural integrity of elastin fibers in spleen [30].

### Disproportionate Abundance

Genes with disproportionate abundance in cliques from one treatment can also be used as a starting point for gene-centered approaches to extracting biological information. To assess each gene's relative representation in cliques in each condition we assigned a scaled difference score (SDS). SDS was calculated as the difference in clique membership between control and IR, expressed as a percentage to correct for different clique numbers between the two conditions, and scaled between 0 and 1 with higher scores indicating a greater difference. An SDS of 1 represents the most extreme case, in which a gene is present in cliques in one condition but not in the other. Based on this metric we selected several genes of interest. For example, cytochrome P450-family 2, subfamily s, polypeptide 1 *(Cyp2s1)* exhibited an SDS of 1, present in 0.4% of IR cliques formed through significant edges with 146 other genes. *Cyp2s1* encodes a novel member of the cytochrome P450 (Cyp) family and is abundantly expressed in spleen as well as epithelial tissues [31,32]. Cyp enzymes are well known for their role in oxidative metabolism of endogenous compounds and xenobiotics, and some Cyp family members may also play roles in basic developmental processes [33–35]. GO enrichment analysis indicated that the genes with which *Cyp2s1* shares edges in IR were significantly enriched (*p* = 0.0019) in the functional terms of primary and cellular metabolism, accounting for 33% of *Cyp2s1* partners. Many of the genes within this subgroup (21/43) were annotated with the GO term cellular protein metabolism, consistent with the general function of Cyp enzymes. Therefore, although the functions of *Cyp2s1* in this context are undefined, its tight co-expression with a set of genes only in IR identifies a putative gene network with which it interacts in the response to radiation. Another example of disproportionate abundance is phospholipase C-L2 *(Plcl2),* present in 17.2% of IR cliques but only 0.3% of control cliques. The largest cliques containing *Plcl2* are enriched for genes involved in immune response. *Plcl2* is expressed in hematopoietic cells and encodes a novel phospholipase C-like protein that lacks lipase activity and instead regulates B-cell receptor signaling and immune responses [36], consistent with the general pathways that were altered in irradiated mice.

Both individual genes and subsets of genes may show disproportionate abundance in cliques of one treatment compared to another. In other words, a set of genes may consistently appear together in cliques of IR but not control, suggesting that these genes might represent the core of a pathway that is treatment-specific. We identified a set of seven genes that co-appeared in many cliques in IR but not control; four of the seven were differentially expressed after radiation. More specifically, 12,238 (~1.1%) cliques in IR contained at least five of these genes. In controls, no more than two of these genes appeared together, and those pairwise interactions were limited to only two combinations, representing 0.07% of cliques in the graph. The core included TGF-β, inducible form *(Tgfbi),* signal transducer and activator of transcription 1 *(Stat1),* sperm mitochondria-associated cysteine-rich protein *(Smcp),* the variable regions of two antibodies (Ig active kappa-chain mRNA V-region and anti-DNA antibody kappa light chain variable region), transmembrane protein 65 *(Tmem65),* and tubby-like protein 4 *(Tulp4).* Of this set, *Tgfbi, Stat1,* and the anti-DNA antibody have clear links to radiation exposure and its potential consequences. *Tgfbi* encodes a secreted adhesion molecule whose expression is sharply induced by TGF-β [37], a protein that senses oxidative stress and orchestrates the response to the DNA-damaging effects of ionizing radiation [28]. Stat1 integrates signal transduction and transcriptional responses to cell stressors, including ultraviolet B radiation, inflammation and infection [38]. Anti-DNA antibodies are activated by reactive oxygen species, which are byproducts of the hydrolysis of intracellular water by IR. The ensuing free radicals cause oxidative damage to DNA, and DNA modified in this way becomes highly immunogenic, activating the production of antibodies directed against it [39]. Among those with no direct link to radiation, Mcsp is a structural protein of mitochondria that has been characterized for its role in sperm motility [40]. However it is also relatively highly expressed in spleen (UCSC Mouse Genome Browser, Aug. 2005 build), where its function is unknown to date. No functional information is available for *Tmem65.* However given its repeated and tight associations with the other genes discussed here, Tmem65 may also have an important role in the response to IR, a possibility that could now be pursued experimentally. Tulp4 is an uncharacterized member of the tubby superfamily of proteins, all of which share the tubby signature motif, nuclear localization signals and suppressor of cytokine signaling domains [41]. Co-expression between all seven members of this gene set in IR but not control suggests that they may function in the same or intersecting pathways in the radiation response, a possibility worth further exploration.

**Figure 6:**
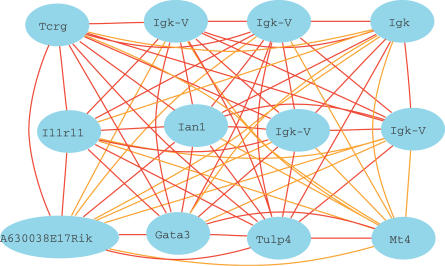
Genes Co-Expressed with Tulp4 in HSCs Gene expression data from HSCs [43] were used in WebQTL (webqtl.org) to identify genes most highly correlated with *Tulp4*. The majority of genes encode proteins involved in immune function (e.g., immunoglobulins).

In addition to the six genes with which it often shares cliques, *Tulp4* was abundant in cliques enriched for immune response genes. To determine if there was independent evidence from other data linking *Tulp4* with the immune system, we identified genes highly correlated with *Tulp4* in an independent set of gene expression data collected from hematopoietic stem cells. WebQTL (http://www.webqtl.org) is an internet resource that serves as a data repository and analysis engine for physiological, microarray and proteomic data collected across several recombinant inbred panels of rodents [42]. Included within WebQTL is a set of microarray expression data collected from hematopoietic stem cells (HSCs), a cell type enriched in spleen [43]. We used the analytical tools within WebQTL to identify genes highly correlated with *Tulp4* in the HSC dataset. Most genes significantly correlated (*p* < 0.000001) with *Tulp4* in HSCs were related to immune function. The 11 most highly correlated (r > 0.77) included five immunoglobulin segments, a T-cell receptor and interleukin 1 receptor-like 1 *(Il1rl1);* all are displayed as a network graph in Figure 6. Although unproven experimentally at this point, these data from an external source conceptually validate the hypothesis that *Tulp4* plays an as yet undefined role in immune function. Given that *Tulp4* is upregulated after IR and that it encodes a putative transcription factor, it is possible that is plays a role in orchestrating the immune response to radiation exposure. These data illustrate that relationships highlighted by clique membership can be independently supported using other datasets and tools and highlight how this approach can be used to filter genes worthy of further experimental study.

### Edge Level Comparisons—Differential Correlations

**Figure 7:**
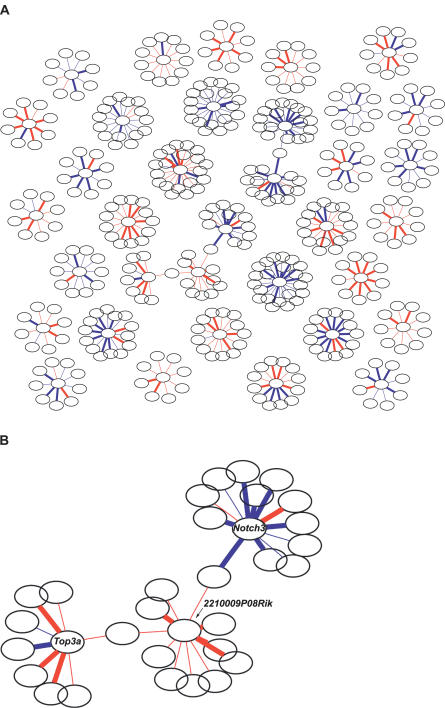
Differential Correlation Identifies Edges Impacted by IR The graph was filtered to identify edges that exceeded r = |0.875| in one condition but were less than |0.25| in the other. Vertices with > 8 differential correlations are represented in (A). Red indicates edges that are present only in IR, while blue edges are only found in control. Dark edges for each color represent the subset of edges that are differentially correlated and of opposite direction (+ vs. −) in the two conditions, while bright edges are of the same direction. The portion of the graph containing three connected sub-graphs centered around *Top3a*, *Notch3,* and an unannotated gene is shown in (B).

The next level of queries focused on what we term differential correlations, i.e., significant gene-gene relationships (edges) found in one condition but not another. Differential correlation provides a means to identify edges that are dramatically altered by treatment and that would not be recognized by examining cliques alone. We defined a differential correlation as an edge that was present above the specified 0.875 threshold (| r| > 0.875) in one condition (control or IR) and for which the corresponding correlation value in the other condition was less than 0.25 (| r| < 0.25). Figure 7 illustrates the results of differential correlation analysis. An overall representation is depicted in Figure 7A, containing vertices with eight or more differential edges. These graphs represent a way to visualize sets of edges activated or repressed by IR that are centered around a single gene. For example, Figure 7B illustrates the differential edges linked to topoisomerase III alpha *(Top3a)*. Unlike other members of the topoisomerase family, Top3a has poor DNA helicase activity and instead appears to interact with RecQ helicases to maintain genomic stability [44]. Top3a associates with Bloom protein, a RecQ family member that participates in cell cycle checkpoint control after exposure to IR [45,46]. Although there are relatively few negative edges in the graph, all of the differential edges connected with *Top3a* are negative, reflecting a set of inverse relationships appearing only in mice from the IR group. The network of genes around *Top3a* is connected to two other structures in the graph centered around an uncharacterized gene (2210009P08Rik) and *Notch3,* which encodes a signaling protein crucial for T cell development [47]. Further study will be necessary to determine if these three sets of genes interact in a functional way in the radiation response.

### Multilevel Criteria for Identifying Genes for More Detailed Study

We used a triplet of criteria to highlight genes that should be prioritized for further study, based on their responses to radiation and on their presence in dense sub-graphs. Specifically, we identified a set of genes that were 1) differentially expressed after irradiation in at least one strain of mice, based on a mixed model analysis and *p* <0.05, 2) differentially correlated, participating in at least one edge that was activated/repressed by radiation (| r| > 0.85 / | r| < 0.25), and 3) differentially abundant, exhibiting a scaled difference score > 0.65. We refer to this approach as the triple screen. A total of 114 genes met all three criteria. We then used GO analysis to determine if these genes that appear to play significant roles in the radiation response were enriched in any functional categories. GO annotations within the category of biological process existed for 43 (of 114) genes. This subset of 43 was significantly enriched (*p* = 0.020) in genes annotated with the GO term “negative regulation of physiological processes.” Three of the four genes in this category were associated with “regulation of apoptosis,” a known response to radiation. The category “response to stress” was also significantly overrepresented (7/43) among this group of genes, reflective of the stress response induced by radiation exposure [48]. The triple screen illustrates how graph structures can be combined with differential expression analysis to highlight sets of genes that respond collectively to IR. The genes identified represent an interesting set of targets to mine for further study of the effects of IR in spleen.

## Discussion

Microarrays represent an incredibly powerful tool to identify sets of genes that respond to condition(s) of interest and underlie biological responses. Both exciting and often frustrating is the sheer volume of data that arrays produce. Even as few as 20–40 differentially expressed genes may be difficult to filter through to select a limited number of candidates for further experimental study, particularly when the goal is validation with in vivo models. Another challenge is that, despite complete sequencing of many genomes, many genes highlighted as interesting have little or no functional annotation, limiting the biological insight that results from their changes in expression. The concept of guilt-by-association, annotating functions of unknown genes based on their co-expression with better-characterized partners, forms the basis for a broad range of clustering methods designed to group genes based on similarity in expression level [2]. A number of recent analyses of large scale expression datasets have validated the concept by mapping correlated gene sets onto GO annotation as a surrogate for gene function. GO represents the best available systematic method for gene functional assignments, although context-specific actions of some proteins prevent the annotations from being comprehensive. Using variations on this strategy, Zhang et al. [49], Wolfe et al. [1], Lee et al. [50], and Stuart et al. [4] all have reported that gene co-expression is a compelling indicator of gene function. These studies validate the rationale for grouping genes by co-expression as a means to expand biological insight provided by array data.

Many algorithmic approaches to guilt-by-association have been applied, and all begin with a measure of similarity (e.g., Euclidean distance, Pearson's coefficient, etc.) calculated for all possible pairwise combinations of expression values followed by an algorithm to organize genes into clusters in a supervised or unsupervised fashion [7,51]. Although they are used almost universally in analysis of microarray data, most clustering algorithms share several limitations. The main caveat is that they are disjoint, assigning a gene to only one cluster. In reality, many genes (proteins) participate in multiple pathways that may have little functional overlap, and examples of such functional diversity abound in the literature [52–54]. Hierarchical clustering methods also do not recognize inverse relationships between genes, which are of equivalent biological interest. Finally, the interpretation is largely visual, based on recognizing patterns displayed in dendograms.

Relevance networks for microarray data are similar to common clustering methods in that both are based upon some measure of similarity between gene expression [9]. They differ in that genes are analyzed for co-expression only after restricting the correlation matrix to edges exceeding a threshold selected to represent biologically meaningful co-expression. To date, applications of relevance networks have been limited by the ability to extract embedded relationships from within the graphs. Previous reports of their use have relied on either identifying pairwise interactions between genes or graphically displaying the entire set of nodes and edges that remain in the graph [4,10–12]. For cases in which the matrix was limited to only genes of interest, the resulting graphs were small and tractable using these methods [11,12]. When the correlation matrix is created from genome scale expression data, however, additional measures are needed to identify co-expressed genes. Our approach was developed as a means to extend beyond pairwise interactions and to identify all complete sub-graphs (cliques) from within a relevance network graph. Clique is widely known for its application in a variety of combinatorial settings, a great number of which are relevant to computational molecular biology [55]. It is particularly useful in microarray analysis, because it addresses the previously-noted shortcomings of traditional clustering algorithms. A vertex can be in more than one clique, and negative correlations are included by temporarily taking the absolute value of correlation coefficients just prior to thresholding. Solving clique is a major computational bottleneck, however, and a classic graph-theoretic problem in its own right [13]. We applied novel graph algorithms that allowed us to compute clique efficiently by employing fixed parameter tractability and focusing on clique's complementary dual, the vertex cover problem [14–17]. These algorithms were used to extract all complete sub-graphs present in the graph, which was created by thresholding the correlation matrix at r >|0.875| and which included only a very small percentage of all possible edges (0.061% control; 0.068% IR). Again, in contrast to clustering methods, this insured that only gene-gene interactions of a specified strength were identified as co-expressed.

Selection of an appropriate threshold value is an important issue for this approach, and there is little experimental data on which to base the selection. Allocco et al. [9] previously analyzed microarray data from several hundred hybridizations across multiple conditions in yeast and related co-expression to the presence of shared transcription factor binding sites as an index of co-regulation. They reported that 50% of gene pairs with r > 0.84 were likely to be co-regulated (not just co-expressed) if a sufficient number of hybridizations were analyzed. We used a value slightly more restrictive than 0.84 to account for the reduced number of arrays analyzed in our example. Other applications of relevance networks arbitrarily have selected a similar but somewhat lower value of 0.8 [11,12]. Moriyama et al. [10] used permutations to identify correlation threshold values with increasing confidence levels (*p* < 0.05, 0.01, 0.001), applying these cutoffs to relevance networks of chemosensitivity and gene expression [10]. Based on Fisher's z-transformation and Bonferroni correction for multiple testing, our threshold of 0.875 produced an effective *p* = 0.0013, indicating a statistically significant level of confidence in relationships represented in the control and IR graphs.

Differential clique analysis can be used to ask a variety of questions from the data, several of which we have illustrated using the response to low-dose IR. Our results with *Ltbp2* and *Plcl2* demonstrate using guilt-by-association to learn more about a gene based on its treatment-specific co-expression patterns. Although both of these genes are members of well-studied gene families (TGFβ-binding proteins and phospholipase enzymes, respectively), each has been suggested to be a novel member of its respective family, with atypical functions [56,57]. Therefore the co-expression profiles identified in response to IR suggest biological roles for each gene that would not be revealed based on structural similarity to known proteins or on sequence conservation. Differential clique analysis can also used to identify core sets of genes that appear together in a condition-specific manner, as illustrated by the co-abundance of the set of seven genes including *Stat1* and *Tulp4* in IR but not control. This core includes two predicted transcription factors *(Stat1* and *Tulp4),* two members of a class of genes (immune responders) differentially expressed in our model, two genes *(Tmem65, Mcsp)* for which there is little or no information about their role in spleen biology, and *Tgfbi,* a gene induced by a key player in the radiation response (TGFβ). The next step will be to determine if these genes are not just co-expressed but co-regulated, potentially through common regulation by Stat1 and Tulp4. Co-expression of *Tulp4* with immune genes in a completely independent dataset from a comparable biological sample (HSCs) further supports this possibility.

Many issues can be further developed to improve this approach. Systematic and statistical approaches to compare clique membership between conditions will improve the iterative process we presented here. Although an advantage of clique is that it is not disjoint, this also creates a high degree of overlap between cliques that complicates the analysis. Similarity metrics that merge overlapping cliques into metacliques are under development. Solving clique also relies on an edge meeting the defined threshold. As a result, edges that fall just short of that value (e.g., r = 0.875 in our dataset) are excluded from the graph, even though they may represent correlations of biological significance. Setting thresholds based only on statistical criteria may result in excluding biologically relevant genetic relationships in small experiments, or including irrelevant relationships in large experiments due to statistical power. We are exploring alternatives, but it is likely that a combination of threshold setting methods will be needed to correctly address statistical and biological concerns. Another consideration is that low thresholds produce a highly connected graph with many vertices, and the computational burden can become unmanageable. We recently described an algorithm we refer to as paraclique that begins to address some aspects of the thresholding problem [58]. Paraclique works by iteratively adding in edges that fall just shy of the original threshold value but still meet user-defined criteria for acceptance. The result are *k*-dense (but not complete) sub-graphs that are more inclusive than those that result from clique alone. We also want to point out that, although the low-dose study was limited to measures of gene expression, almost any type of quantitative data can be included in the correlation matrix. For example, if the current study had included systemic parameters relevant to the immune response, relationships between genes and functional measures (e.g., T-cell numbers) could have been extracted directly, rather than inferred from the data. Future efforts will be directed toward this application.

**Figure 8:**
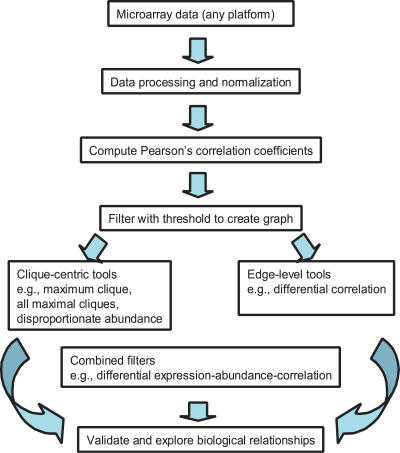
Overall Schema of Our Approach

The complete schema of our method is summarized in Figure 8. Our approach offers the microarray user at least three potential applications: 1) annotation of poorly described genes based on guilt-by-association in a way that permits multiple functional assignments; 2) prioritization of genes for biological validation based on both differential expression and enriched connectivity; and 3) identification of relationships between genes that are differentially activated in a specific condition, rather than just differential expression of individual genes. The latter use may prove to be especially useful for conditions in which a number of genes change in parallel, but few or none of the changes are marked enough to meet statistical criteria for differential expression. For example, Mootha et al. [59] associated coordinate changes in expression of a group of functionally related genes with diabetes. None of the genes were significantly altered individually, with only ~20% differences in expression in skeletal muscle of diabetics compared to healthy controls. However when changes in expression were analyzed in concert, a group of genes was identified that not only correlated with the diabetic phenotype but signaled metabolic alterations in the prediabetic state. By comparison, clique extraction represents a potential means to identify such subsets de novo, without a priori knowledge of the genes that might be involved. Ideally, clique extraction would be followed by validation using an independent set of data to determine if the same pathways could be identified in a replicate experiment. In particular, it is important to determine if some or all of the pathways described herein based on GO enrichment are robust to genetic variation or are unique to the six inbred strains used in this study. For example, if we exposed another set of six different inbred strains of mice to low-dose radiation, would cliques be enriched for the same functional pathways described herein? An ideal population in which to validate findings in this manner would be use of recombinant inbred strains of mice, such as the BXD RI strains created from C57BL6/J and DBA/2J parental strains [60]. The large numbers of strains in RI panels (80 for BXD) create the opportunity to extract pathways across a significant spectrum of genetic variation, for example based on 40 BXD strains, and then validate findings in an equally large subset.

In conclusion, we have described a method to extend the utility of relevance networks by computationally extracting dense sub-graphs of tightly interconnected genes. This furthers the effort to identify gene networks from microarray data and potentially other types of data based on the concept of guilt-by-association. Once created, the graph can be probed in many ways to identify potentially meaningful relationships between genes and sets of genes. Ongoing efforts are directed toward refined methods for selecting a meaningful threshold and incorporating data of multiple types in the graphs.

## Materials and Methods

### Animals and tissue collection.

All mice were bred at Oak Ridge National Laboratory and experiments were conducted under approved Institutional Animal Care and Use Committee protocols. Six standard inbred strains of mice (C57Bl6/J, Balb/C, DBA/2J, A/J, C3H/HeJ and B6.C) 8–10 wk of age were exposed to an acute 10 cGy dose of a broad-spectrum X-ray flux produced by a standard bremsstrahlung source (maximum voltage = 250 kVp, maximum current = 10 mA, filter = 0.2 mm Cu). Only males were used, and each group (control and exposed) consisted of 4–8 mice. Mice were sacrificed 3.5 hr after exposure and tissues were harvested into RNALater (Ambion, The Woodlands, Texas, United States) and stored at −20 °C until RNA isolation.

### RNA and microarrays.

Microarrays representing ~15,000 unique mouse genes were printed by the Center for Applied Genomics (PHRI, Newark, New Jersey, United States) using the Compugen Mouse OligoLibrary (2.0). After printing, slides were air-dried and the cDNAs irreversibly immobilized by UV-crosslinking. Spot quality was assessed by hybridization with fluorescently-labeled panomers according to manufacturer's protocols (Molecular Probes, Carlsbad, California, United States).

Total RNA was isolated from spleen using the RNeasy midi RNA isolation system (QIAGEN, Valencia, California, United States), including a DNAse I treatment step to eliminate contaminating genomic DNA. RNA quality was assessed by visualization in denaturing agarose gel electrophoresis and spectrophotometrically by the 260 nm/280 nm ratio of absorbance. Samples were quantified spectrophotometrically based on the absorbance at 260 nm. Only RNA samples of high quality were used for further analysis.

Total RNA (10 μg) from spleen was fluorescently labeled and hybridized using standard labeling and hybridization protocols [61]. Dye incorporation and labeled cDNA yield were measured by scanning spectrophotometry and calculated from the absorbance values at 260 nm (cDNA) and at either 550 nm (Cy3) or 650 nm (Cy5). Each hybridization consisted of a pair of RNA samples from control and IR-exposed mice (a biological replicate); animals were paired randomly. A dye swap was performed for each biological replicate to control for dye-specific bias in labeling and to provide a replicate hybridization for each pair of samples. A total of at least three biological replicates were analyzed for each inbred strain. Data were normalized using Lowess to adjust for intensity-dependent dye bias after removing spots of poor quality or low expression and subtracting local background [62]. Differentially expressed genes were identified using mixed model ANOVA performed in SAS (Cary, North Carolina, United States) as described by Wolfinger [63] and using a 95% false discovery rate protected confidence interval. Because control and treatment data are treated separately in correlation analysis, data for this application were also normalized between slides by median centering to control for technical variation between hybridizations. Due to the incorporation of a dye swap in the experimental design, two replicate measures of expression existed for each animal, and the normalized values were averaged to produce one measure of expression per animal. Normalized data were used to calculate Pearson and Spearman correlations. Entrez Gene IDs are included parenthetically, as available, for each gene mentioned in text.

### Graph algorithms.

The matrix of Pearson's correlation coefficients from all data meeting criteria for quality and expression level was converted into an unweighted graph. Only genes with observations in at least 19 hybridizations were retained as vertices; only gene pairs whose correlation coefficients were at least |0.875| were included as edges. We employed principles of fixed parameter tractability [64,65] to extract vertex covers, and from them cliques. Thus we reduced problem size using kernelization and searched the resultant kernel efficiently with branching. A complete description of our algorithms can be found in [14,15]. Source codes are freely available from M. A. Langston or any co-author. Our algorithms have also been installed in Clustal XP, a high-performance, parallel version of the Clustal W package (http://ClustalXP.cgmlab.org).

Differential abundance of genes in cliques was determined based on the SDS, which was calculated for each gene based on the percentage of clique membership for each condition (control and IR) and then scaled between 0 and 1. Per cent IR and %control represent the total percentage of cliques in which each gene has membership in each condition. First the relative difference in clique membership between IR and control was calculated as %difference = | (%IR-%control)/((%IR+%control)/2)| * 100. This value was scaled between 0 and 1 by normalizing to the most extreme difference across the entire gene set for which a gene was present in at least one clique from each condition (SDS = (%diff - min %diff) / (max %diff - min %diff)).

Differential correlation describes marked edge level differences in correlation between a pair of genes in control and IR. Herein, a gene is defined as differentially correlated if the following statement is true: r > |0.875| _dose,control_ AND r <|0.25|_control, dose_. Differential correlation graphs were created using GraphViz (2.6).

### Gene ontology enrichment.

Analyses of overrepresentation of GO categories within the ontologies of Biological Process and Molecular Function across the set of differentially expressed genes was conducted using the Database for Annotation, Visualization and Integrated Discovery 2.1 (DAVID 2.1, http://apps1.niaid.nih.gov/David) [66]. The detailed protocols and primary data from this study will be available through the Gene Expression Omnibus database (GEO; http:/www.ncbi.nlm.nih.gov/geo).

## Supporting Information

Figure S1 Diagram of the Experimental Design for the Radiation Exposure and Microarray Hybridizations (14 KB GIF)

Table S1 Complete List of Genes Identified using the Triple Screen (133 KB DOC)

### Accession Numbers

The Entrez Gene (http://www.ncbi.nlm.nih.gov/entrez) ID numbers for the genes and gene products discussed in this paper are *Cyp2s1* (74134), *Ltbp2* (16997), *Notch3* (18131), *Plcl2* (224860), *Smcp* (17235), *Stat1* (20846), *Tgfbi* (21810), *Tmem65* (74868), *Top3a* (21975), *Tulp4* (68842).

## Abbreviations

Cyp: cytochrome p450 family
Cyp2s1: cytochrome P450, family 2, subfamily s, polypeptide 1
GO: Gene ontology
HSCs: hematopoietic stem cells
Il1rl-1: interleukin 1 receptor-like 1
IR: ionizing radiation
Ltbp2: latent transforming growth factor beta binding protein 2
Plcl2: phospholipase C-L2
SDS: scaled difference score
Smcp: sperm mitochondria-associated cysteine-rich protein
Stat1: signal transducer and activator of transcription 1
TGF-β: transforming growth factor beta
Tgfbi: TGF-β, inducible form
Tmem65: transmembrane protein 65
Top3a: topoisomerase III alpha
Tulp4: tubby-like protein 4
