# Supplementary material for: Extracting Gene Networks for Low-Dose Radiation Using Graph Theoretical Algorithms
Source: PLoS Comput Biol. 2006 Jul 21;2(7):e89. doi: 10.1371/journal.pcbi.0020089 (PMC1513268; doi:10.1371/journal.pcbi.0020089)
Supplement: Supplementary file 1 [file pcbi.0020089.st001.doc]

Table S1.

| ENTREZ GENE ID | | GENE NAME |
| --- | --- | --- |
| **11781** |  | **adaptor-related protein complex AP-4, mu 1** |
| NA |  | anti-Lewis Y antigen monoclonal antibody variable heavy chain [mice, mRNA Partial, 357 nt]. |
| **27425** |  | **ATP synthase, H+ transporting, mitochondrial F0 complex, subunit g** |
| **12049** |  | **Bcl2-like 10** |
| **66653** |  | **BRF2, subunit of RNA polymerase III transcription initiation factor, BRF1-like** |
| 76809 |  | Bri3 binding protein |
| **12555** |  | **cadherin 15** |
| 234671 |  | carboxylesterase 2 |
| **70233** |  | **CD2 antigen (cytoplasmic tail) binding protein 2** |
| 228576 |  | cDNA sequence BC012256 |
| **238690** |  | **cDNA sequence BC062958** |
| **20293** |  | **chemokine (C-C motif) ligand 12** |
| **13215** |  | **defensin beta 2** |
| **50768** |  | **deleted in liver cancer 1** |
| 218850 |  | DNA segment, Chr 14, Abbott 1 expressed |
| 170765 |  | Down syndrome critical region homolog 6 (human) |
| **13628** |  | **eukaryotic translation elongation factor 1 alpha 2** |
| 14038 |  | extracellular proteinase inhibitor |
| **64095** |  | **G protein-coupled receptor 35** |
| **93690** |  | **G protein-coupled receptor 45** |
| **74577** |  | **galactosidase, beta 1-like** |
| **14568** |  | **guanosine diphosphate (GDP) dissociation inhibitor 2** |
| **14913** |  | **guanylate cyclase activator 1a (retina)** |
| **66044** |  | **histidyl tRNA synthetase 2** |
| **16149** |  | **Ia-associated invariant chain** |
| 16062 |  | immunoglobulin heavy chain (J606 family) |
| **16162** |  | **interleukin 12 receptor, beta 2** |
| **17105** |  | **lysozyme** |
| NA |  | M.musculus WM65 immunoglobulin gamma one mRNA. |
| **20288** |  | **macrophage scavenger receptor 1** |
| NA |  | Mouse DNA, heavy chain of a recombinant single chain Fv against the human C3a receptor. |
| NA |  | Mouse hybridoma Ig rearranged kappa-chain mRNA V-region, partial cds. |
| NA |  | Mouse Ig active H-chain mRNA, V-region, subgroup IIIB, clone L36. |
| NA |  | Mouse Ig kappa-4-chain mRNA, V-J-region, partial cds, subgroup 2. |
| NA |  | Mouse mRNA for light chain of catalytic antibody 3G6 with hydrolytic activity of chloramphenicol monoester, V region. |
| NA |  | Mouse Q5 class I MHC gene (exon 5 ). |
| NA |  | Mouse T-cell receptor rearranged alpha-chain gene V-alpha-10-J-alpha-26 region. |
| NA |  | Mus musculus 2F2 monoclonal antibody kappa light chain variable region, (IgK) mRNA, partial cds. |
| NA |  | Mus musculus 42F1 immunoglobulin heavy chain variable domain mRNA, partial cds. |
| NA |  | Mus musculus anti-DNA autoantibody G5-8 heavy chain variable region mRNA, partial cds. |
| NA |  | Mus musculus anti-DNA immunoglobulin light chain IgG, antibody 423p.153, partial cds. |
| NA |  | Mus musculus anti-DNA immunoglobulin light chain IgM mRNA, antibody 452p.109, partial cds. |
| NA |  | Mus musculus Biozzi anti-diuron Ig kappa light chain mRNA, partial cds. |
| NA |  | Mus musculus clone A3.c immunoglobulin heavy chain VDJ region gene, partial cds. |
| NA |  | Mus musculus clone N1.1.a immunoglobulin heavy chain VDJ region gene, partial cds. |
| NA |  | Mus musculus F2.3D12 immunoglobulin heavy chain mRNA, partial cds. |
| NA |  | Mus musculus hybridoma 4F10 Ig heavy chain mRNA, partial cds. |
| NA |  | Mus musculus Ig active kappa-chain mRNA V-region. |
| NA |  | Mus musculus Ig rearranged anti-Sm hybridoma mRNA V-region sequence. |
| NA |  | Mus musculus Ig rearranged H-chain (S107 family) mRNA V11-J4 region. |
| NA |  | Mus musculus infected mouse B seq 2, day 10, T cell receptor beta chain mRNA, partial cds. |
| NA |  | Mus musculus isolate O2F3VL immunoglobulin kappa chain variable region mRNA, partial cds. |
| NA |  | Mus musculus JL1/11-7 rearranged immunoglobulin heavy chain variable region mRNA, partial cds. |
| NA |  | Mus musculus mRNA for anti-c-erbB-2/p185 monoclonal antibody SER4 light chain variable region, partial cds. |
| NA |  | Mus musculus olfactory receptor M50 gene, complete cds. |
| NA |  | Mus musculus PDA non-translated mRNA, partial sequence. |
| NA |  | Mus musculus RNA binding site for Dazl protein, clone cf11. |
| NA |  | Mus musculus, Similar to AHNAK-related, clone IMAGE |
| **17188** |  | **MYC-associated zinc finger protein (purine-binding transcription factor)** |
| **71780** |  | **myo-inositol 1-phosphate synthase A1** |
| **17901** |  | **myosin, light polypeptide 1** |
| **170722** |  | **nuclear RNA export factor 7** |
| 231042 |  | nucleoporin like 2 |
| **18351** |  | **olfactory receptor 51** |
| **18613** |  | **platelet/endothelial cell adhesion molecule** |
| **80979** |  | **prestin (motor protein)** |
| **19156** |  | **prosaposin** |
| **20187** |  | **receptor-like tyrosine kinase** |
| **19696** |  | **reticuloendotheliosis oncogene** |
| **19888** |  | **retinitis pigmentosa 1 homolog (human)** |
| **19981** |  | **ribosomal protein L37a** |
| **71699** |  | **RIKEN cDNA 1010001P06 gene** |
| 68509 |  | RIKEN cDNA 1110018H23 gene |
| 68845 |  | RIKEN cDNA 1110061L23 gene |
| **57754** |  | **RIKEN cDNA 1500001H12 gene** |
| 101867 |  | RIKEN cDNA 1500003O22 gene |
| 76392 |  | RIKEN cDNA 1700003F17 gene |
| 69327 |  | RIKEN cDNA 1700007K13 gene |
| 76926 |  | RIKEN cDNA 1700021F13 gene |
| 73429 |  | RIKEN cDNA 1700064D17 gene |
| 73571 |  | RIKEN cDNA 1700096K18 gene |
| 76629 |  | RIKEN cDNA 1700111I05 gene |
| 78481 |  | RIKEN cDNA 1700123J03 gene |
| 66249 |  | RIKEN cDNA 1810003N24 gene |
| 65963 |  | RIKEN cDNA 1810009M01 gene |
| 76942 |  | RIKEN cDNA 2210003I03 gene |
| 76730 |  | RIKEN cDNA 2310005C01 gene |
| 76443 |  | RIKEN cDNA 2310016D23 gene |
| 69627 |  | RIKEN cDNA 2310031A18 gene |
| **66985** |  | **RIKEN cDNA 2400009B11 gene** |
| **72494** |  | **RIKEN cDNA 2610202E01 gene** |
| 67058 |  | RIKEN cDNA 2810428J06 gene |
| 76835 |  | RIKEN cDNA 2900052L18 gene |
| 72971 |  | RIKEN cDNA 2900053O20 gene |
| 75165 |  | RIKEN cDNA 4930535D10 gene |
| 67668 |  | RIKEN cDNA 4930563N14 gene |
| **71130** |  | **RIKEN cDNA 4933424C13 gene** |
| 71205 |  | RIKEN cDNA 4933431M02 gene |
| 71321 |  | RIKEN cDNA 4933440J02 gene |
| 76676 |  | RIKEN cDNA 5330411J11 gene |
| 76191 |  | RIKEN cDNA 6330575B07 gene |
| 71465 |  | RIKEN cDNA 8430413B15 gene |
| 75755 |  | RIKEN cDNA 9030420N05 gene |
| 77667 |  | RIKEN cDNA 9130231A04 gene |
| 19874 |  | RNA, Y3 small cytoplasmic (associated with Ro protein) |
| **20846** |  | **signal transducer and activator of transcription 1** |
| **20527** |  | **solute carrier family 2 (facilitated glucose transporter), member 3** |
| **171429** |  | **solute carrier family 26, member 6** |
| 245865 |  | sperm associated antigen 4 |
| 68416 |  | syncollin |
| **22067** |  | **transient receptor potential cation channel, subfamily C, member 5** |
| **170829** |  | **translocating chain-associating membrane protein 2** |
| **22302** |  | **vomeronasal 2, receptor, 11** |
| 22441 |  | X-linked lymphocyte-regulated complex |

Entries in boldface type represent genes for which GO annotation was available.
